# Intraoperative Awareness and Recall: A Comparative Study of Dexmedetomidine and Propofol in Cardiac Surgery

**DOI:** 10.7759/cureus.1542

**Published:** 2017-08-05

**Authors:** Tufail Ahmad, Nadeem A Sheikh, Nihida Akhter, Bashir A Dar, Riyaz Ahmad

**Affiliations:** 1 Anesthesiology and Critical Care, Sher-i-Kashmir Institute of Medical Sciences Medical College, Srinagar, India; 2 Registrar Internal Medicine, SMHS Hospital, Srinagar; 3 Registrar Obstetrics and Gynecology, LD Hospital; 4 Department of Anaesthesiology, Sher-i-Kashmir Institute of Medical Sciences Medical College, Srinagar, India

**Keywords:** bispectral index, cardiac surgery, dexmedetomidine, propofol, brice questionnaire

## Abstract

Background

Awareness during general anesthesia is undesired and unanticipated patient wakefulness during surgery or recall of intraoperative events. Incidence of awareness in patients undergoing cardiac surgery is significantly higher than the overall incidence of 1% during general surgery. Awareness during cardiac surgery can be prevented by a number of methods. One such method is the supplemental, intraoperative use of sedative agents. Propofol, a bisubstituted phenol, is an intravenous general anesthetic that has been shown to reduce the incidence of awareness. Dexmedetomidine—an alpha_2_-adrenergic agonist with anxiolytic, opioid, and general anesthetic-sparing properties—is being considered for maintaining intraoperative depth of anesthesia. The purpose of this study was to evaluate the effect of dexmedetomidine on depth of anesthesia and to compare it with the effect of propofol in cardiac surgery.

Methods

This was a prospective, randomized, double-blind study conducted in a tertiary-care hospital. Sixty patients with American Society of Anesthesiologists (ASA) physical status I-III planned for elective open heart surgery were randomized into two groups of 30 patients each. Each patient of the dexmedetomidine group received an initial loading dose of dexmedetomidine at 1 mcg kg^-1^ over 10 minutes followed by infusion at the rate of 0.2–0.6 mcg kg^-1^ hr^-1^. Patients of the propofol group received propofol infusion at the rate of 0.25^-1^ mg kg^-1^ hr^-1^. An identical technique—of standard general anesthesia and routine physiological monitoring—was used in both groups. Bispectral scores were recorded at predetermined intervals during surgery and the target bispectral index (BIS) was kept at 50±10. The patients were assessed for awareness and recall 24 hours after tracheal extubation using the Brice Questionnaire.

Results

Intraoperative BIS scores remained within the target range in both groups; however, the BIS scores showed variable trends between the groups and were significantly lower in the dexmedetomidine group (p < 0.001). None of the patients in either group had recall of intraoperative events.

Conclusion

Administration of dexmedetomidine was as effective in reducing awareness and recall in cardiac surgery compared to propofol. Thus, dexmedetomidine can be used as an alternative sedative agent to prevent awareness and recall in cardiac surgery.

## Introduction

Many patients facing surgery dread the prospect of being awake, in pain, and unable to move owing to inadequate general anesthesia. Awareness during anesthesia with subsequent explicit recall is distressing and may contribute to posttraumatic stress disorder (PTSD) [1-3]. The incidence of intraoperative awareness during general surgery, as reported in the literature, varies between 0.1% and 0.9% [4]. Incidence of awareness in patients undergoing cardiac surgery is significantly higher, with older reports of up to 23% [5].

Several monitors, based mostly on processed electroencephalograph (EEG) information or auditory evoked potentials, have been developed in an attempt to measure depth of anesthesia [6]. It is hoped that the use of such monitors during general anesthesia will decrease the likelihood of awareness. Of these monitors, the bispectral index (BIS) has been most widely adopted in clinical practice. The BIS monitor incorporates a proprietary algorithm that analyzes signals from a processed scalp EEG. The monitor shows a dimensionless number between 0 and 100, with lower numbers reflecting deeper anesthesia [7-8].

Propofol, a bisubstituted phenol, is an intravenous general anesthetic that is given to patients for titratable sedation and hypnosis due to its quick onset and offset [9]. A continuous infusion of the standard rate of propofol has been shown to reduce the incidence of awareness [10]. Dexmedetomidine, an alpha2-adrenergic agonist, has been approved for use as a sedative-analgesic. It has anxiolytic, sympatholytic, opioid, and general anesthetic-sparing properties [11]. However, little—if any—information has been published on the effect of dexmedetomidine on the depth of anesthesia in cardiac surgery. The aim of this study was to evaluate the effect of dexmedetomidine on depth of anesthesia and compare it with that of propofol by using BIS values and to study the feasibility of dexmedetomidine and propofol as sedative agents in maintaining depth of anesthesia and in preventing intraoperative awareness.

## Materials and methods

This study was conducted at the Department of Anesthesiology at Sher-i-Kashmir Institute of Medical Sciences, Srinagar, after receiving the approval of the institutional ethical committee. The study included 60 patients aged 15–60 years with American Society of Anesthesiologists (ASA) physical status I-III and planned for elective open heart surgery under general anesthesia. The study was conducted in a prospective, randomized, double-blind manner. Proper informed consent was taken from all patients included in the study.

Patients having neurological/psychological disorders, renal/hepatic dysfunction, or allergy to propofol or dexmedetomidine were excluded from the study, as were patients on antipsychotics or sedatives and those belonging to New York Heart Association (NYHA) class IV (left ventricular ejection fraction less than 40%). Patients with infusions of catecholamines or vasodilators before anesthesia were also excluded.

Patients were randomly allocated to two groups—dexmedetomidine and propofol—of 30 each, using computer-generated random numbers in sealed envelopes. Patients were premedicated with oral diazepam 5 mg the night before surgery. Intravenous access was obtained in the operating room. Routine physiological monitoring included electrocardiography (ECG), pulse oximetry, non-invasive blood pressure, temperature and neuromuscular monitoring, urine output, and BIS. Baseline haemodynamic variables were recorded. BIS was obtained using disposable sensors (Aspect Medical Systems, Inc., Norwood, MA). EEG electrodes were placed in a bifrontal montage after skin preparation with disinfectant alcohol and slight rubbing. The EEG signal acquired and the BIS displayed on the monitor (Mindray WATO EX-65, China) were recorded. Patients were induced with propofol (1–2.5 mg kg-1), morphine (0.1–0.2 mg kg-1), isoflurane (minimum alveolar concentration 1.2), and vecuronium (0.1–0.2 mg kg-1) for tracheal intubation. The dexmedetomidine group received a bolus dose of dexmedetomidine (1 mcg kg-1 over 10 minutes), followed by infusion (0.2–0.6 mcg kg-1 hr-1). The propofol group received a propofol infusion at the rate of 0.25-1 mg kg-1 hr-1. Target BIS was kept at 50±10. The lungs were ventilated with 100% oxygen using intermittent positive pressure ventilation (IPPV). Tidal volume and respiratory rate were adjusted to maintain an end tidal carbon dioxide rate of 30–35 mmHg. A central venous catheter and arterial cannula were inserted subsequently for transduction of central venous pressure and invasive blood pressure. Anesthesia was maintained with isoflurane and intermittent additional boluses of 0.05 mg kg-1 morphine were administered before skin incision and sternotomy, at commencement, and toward the end of cardiopulmonary bypass (CPB). Muscle relaxation was maintained with vecuronium bromide (0.01–0.02 mg kg-1). Heart rate and mean arterial pressure were kept within 25% of the baseline. Tachycardia and hypertension were treated with esmolol and nitroglycerine as appropriate. Hypotension was treated with fluid boluses, phenylephrine, and inotropes, as appropriate. The Maquet HL20 heart lung machine (MAQUET, Germany) and membrane oxygenator were used during conduct of CPB. After the aortic root had been cannulated, the necessary cannulae were placed and moderate hypothermic CPB (28–32°C) was initiated. While patients were on full CPB flows, lungs were not ventilated. Mean perfusion pressure was maintained between 50–80 mmHg during CPB and any deviation was treated with nitroglycerine or phenylephrine boluses as appropriate. Myocardial protection was achieved with cold potassium-enriched blood cardioplegia solution and topical application of cold saline and ice slush. Patients’ temperature was regulated using a heat exchanger. The fresh gas flow was adjusted to maintain the normal acid-base balance using the alpha stat strategy and rewarming was performed to 37°C. At our institute, it was neither usual nor feasible to add isoflurane to the CPB circuit; therefore, isoflurane was used before and after CPB and infusion of propofol or dexmedetomidine was continued during CPB.

BIS scores were recorded during surgery at the following time points: prior to induction; post-intubation; at sternotomy; before CPB and five minutes after onset of CPB; before and after aortic cross-clamping; every 15 minutes after aortic cross-clamping; after aortic cross-clamp release; rewarming phase of CPB; before and after weaning from CPB; after closure of thorax; and at the end of surgery.

After skin closure, the dexmedetomidine/propofol infusion was stopped. The patients were shifted to the CICU without reversing the muscle relaxation for elective mechanical ventilation, and extubated when standard criteria for weaning and extubation were met. The patients were subjected to a structured interview for awareness and recall 24 hours after tracheal extubation (Brice Questionnaire) as under.

1. What was the last thing you remember before going to sleep for the operation? 2. What was the first thing you remember after waking after the operation? 3. Do you remember anything in between these two periods? 4. Did you have any dreams during your operation? 5. What was the most unpleasant thing you remember from your operation and anesthesia (noises/voices, feeling anything, or waking up)?

If the patient indicated that he or she did not have explicit memory of intraoperative events while answering these questions, no further questions were asked. If the patient indicated that he or she had explicit memory of intraoperative events while answering the questions, the following subquestions were asked:

1. What did you notice: sounds, touch, pain, paralysis?

2. How long did it last?

3. Did you try to alert anyone?

4. Have there been any consequences for you?

Awareness was defined as the presence of explicit memory of any event from induction of anesthesia to recovery of consciousness in the CICU.

Statistical Analysis

Standard statistical tests (​Student’s *t*-test, chi-squared test, and Wilcoxon-Mann-Whitney *U *test ) were used to analyze the data. The results so obtained have been discussed with a 5% level of significance (p value < 0.05) considered significant. SPSS 20 (IBM, NY) for Windows was used to analyze the data.

## Results

Sixty patients were enrolled as per the inclusion criteria. They were randomly divided into two groups—dexmedetomidine and propofol. These two groups were comparable with respect to patient characteristics (Table *1*).

**Table 1:** Patient characteristics Data is expressed as mean+SD or number (%). ASA: American Society of Anesthesiologists NYHA: New York Heart Association LVEF: left ventricular ejection fraction CPB: cardiopulmonary bypass

	Dexmedetomidine group	Propofol group	P value
Age (y)	33.6 + 11.816	35.56 + 9.54	0.48
Weight (kg)	55.5+9.83	53.63+8.31	0.43
Gender (female/male), n (%)	18(60.0)/12(40.0)	22(73.3)/8(26.6)	0.99
ASA status (I/II/III), n (%)	13(43.3)/17(56.7)/0(0.0)	13(43.3)/17(56.7)/0(0.0)	1
NYHA (I,II,III), n (%)	0(0.0)/29(96.7)/1(3.3)	1(3.3)/28(93.3)/1(3.3)	1
LVEF (%)	65.96 + 7.36	64.13 + 4.09	0.23
CPB time (min)	105.43 + 48.69	118.93 + 52.49	0.3
Cross-clamp time (min)	75.2 + 44.66	83.96 + 44.83	0.45

**Figure 1:**
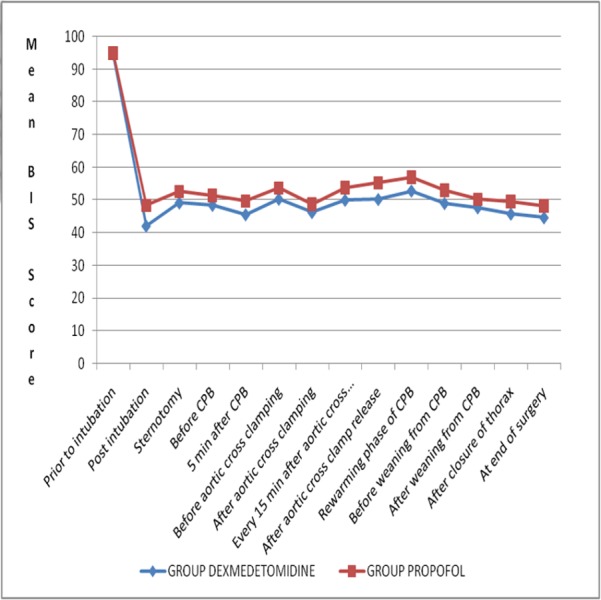
Comparison of BIS scores between study groups The BIS scores remained below 60 in both groups, indicating adequate depth of anesthesia; however, the dexmedetomidine group had significantly lower BIS scores compared with the propofol group (p<0.001).

Intraoperative BIS scores were recorded to monitor depth of anesthesia. Mean BIS scores prior to intubation were 94.76+1.99 in the dexmedetomidine group and 95.10+2.02 in the propofol group. The scores were comparable and statistically insignificant (p=0.523). Post-intubation, and throughout the study period until the end of surgery, BIS scores remained within the target range (40–60). The difference between the BIS scores was statistically significant (p< 0.001). The dexmedetomidine group recorded lower BIS scores compared with the propofol group (Figure* 1*).

Postoperatively, all the patients were interviewed with the same structured modified Brice Questionnaire and the answers were recorded. Twelve (40.0%) patients in the dexmedetomidine group and 14 (46.6%) patients in the propofol group remembered the placement of the intravenous cannula before they were put to sleep. Waking up in the CICU was the first memory after surgery experienced by 14 patients (46.6%) in the dexmedetomidine group and 11 (36.6%) patients in the propofol group. Patients of both groups experienced pleasant dreams; however, the occurrence was statistically insignificant (p value= 0.308). None of the patients had recall of intraoperative events. Nothing in the patients’ answers in the Brice interview indicated awareness during surgery in either group (Table *2*).

**Table 2:** Brice questionnaire None of the answers given by the patients in either study group revealead intra-opearative awareness.

Last thing to remember before operation	Group	
	Dexmedetomidine	Propofol
Being in operation theatre	10 (33.3)	8 (26.6)
Feeling pain from intravenous line	12 (40.0)	14 (46.6)
Supplicating God	3 (10.0)	5 (16.6)
No recall	4 (13.3)	1 (3.3)
First thing you remember after waking up	Group	
	Dexmedetomidine	Propofol
Hearing voices of people around	5 (16.6)	7 (23.3)
Feeling pain	6 (20.0)	9 (30.0)
Seeing place around	14 (46.6)	11 (36.6)
No recall	4 (13.3)	0 (0.0)
Any recall inbetween these two periods	Group	
	Dexmedetomidine	Propofol
	None	None
Dreams	Group	
	Dexmedetomidine	Propofol
Yes	1 (3.3)	3 (10.0)
No	29 (96.6)	27 (90.0)
Worst thing about your operation	Group	
	Dexmedetomidine	Propofol
	None	None

## Discussion

The term ‘awareness’ is limited to ‘explicit memory’ during anesthesia and does not include the time before general anesthesia is fully induced or the time of emergence from general anesthesia [12]. ‘Explicit memory’ is assessed by the patient’s ability to recall specific events that took place during general anesthesia. Many patients may relate that they heard and were aware of everything during a previous anesthetic experience. Affected patients report perception of paralysis, conversations, and surgical manipulations, accompanied by feelings of helplessness, fear, and pain [13].

Cardiac surgical patients might be at higher risk of intraoperative awareness. Reported rates of intraoperative awareness during cardiac surgery range from 0.2% to 2%, a tenfold increase in risk compared with the general surgical population [14-15]. Most cases occur during or after rewarming from CPB and are attributed to alterations in drug pharmacokinetics and dynamics [16]. Patients with impaired cardiac function (e.g., low ejection fraction, pulmonary hypertension) are vulnerable to hemodynamic compromise with anesthetic administration. Practitioners therefore might attempt to minimize anesthetic administration to these patients [17]. The precise concentration of the anesthetic required to guarantee lack of recall is unknown. Reliance on clinical signs is certainly not enough, especially with the use of muscle relaxants that abolish two of the most valuable indicators of depth of anesthesia, i.e., respiration and movement in response to surgery [18]. In the current scenario of cardiac anesthesia, the need for a reliable monitor that ensures unconsciousness is highly desirable. BIS monitoring is used by clinical anesthetists to titrate anesthetic agents in order to maintain loss of consciousness (LOC) and prevent intraoperative awareness [19,7].

The current study revealed no definitive cases of intraoperative awareness. Groesdonk et al. [20] evaluated 514 patients undergoing fast-track cardiac surgery. They recorded only one possible case of intraoperative awareness in more than 510 patients. The patient in question reported an episode of possible intraoperative recall, but this was determined subsequently to have been caused most likely by awakening in the post-anesthesia care unit (PACU). Because the authors could not completely exclude that this patient was aware during the time of surgery, however, the occurrence of awareness in their study was specified as 0.2%.

Dowd et al. [10] studied 617 patients undergoing cardiac surgery. After initiation of CPB, propofol infusion (2–6 mg kg-1 hr-1) was started. The authors reported incidence of intraoperative awareness of 0.3%. The absence of intraoperative awareness in our study may be attributed to continuous administration of drug infusions at all times during surgery, especially during the period of CPB, and the use of a balanced anesthesia technique using inhalational agents and benzodiazepines prior to and after CPB. Dexmedetomidine infusion to prevent awareness was used by Chattopadhyay et al. [21], who studied 60 patients scheduled for laparotomy under general anesthesia. Patients received either propofol 1 mg kg-1 bolus followed by infusion (50 mcg kg-1 min-1) or dexmedetomidine 1 mcg kg-1 bolus followed by infusion (0.5 mcg kg-1 h-1). None of the patients had recall of any intraoperative events.

Although previous authors have acknowledged that the most appropriate method to investigate awareness is to conduct a structured postoperative interview, the actual timing of the interview is a matter of debate. Some advocate that patients be interviewed as soon as they regain consciousness. However, most patients will be drowsy and may therefore give an unreliable interview. Others suggest that patients be interviewed much later, even as long as one week after their operation [22]. This would obviously eliminate patients already discharged from the hospital. An early interview during the first 24 hours is suggested as a reasonable time—thus this was the timing of the postoperative interview in this study [23].

Patients of both the groups had dreams which were pleasant, and the content was unrelated to surgery. In Leslie et al.’s study [24], 300 patients presenting for elective surgery were studied. Patients were interviewed on emergence and 2–4 hours postoperatively. Dream content and form were assessed. Dreaming was reported by 22% of patients on emergence. Leslie et al. concluded that dreaming during anesthesia is unrelated to the depth of anesthesia.

In the present study, BIS readings of adequate anesthetic depth were achieved among both groups at all time points of data collection; however, BIS values were lower in the dexmedetomidine group. Recovery of consciousness during general anesthesia without any recall (in the absence of surgical stimulus) has generally been associated with a BIS value of 60 [25]. Cases of awareness during surgical stimulation with high BIS values (60) have also been reported [26]. Although there is at least one case report of awareness with a BIS of apparently 50 [27], BIS was subsequently found to be 60 at the probable time of awareness [28].

The limitations of the current study include a small sample size and the timing of the postoperative interview, which may have contributed to a falsely low incidence of explicit recall. The amnestic effects of premedication as well as general anesthetics may also have resulted in low incidence of intraoperative awareness.

## Conclusions

The current prospective investigation involved the use of dexmedetomidine to prevent awareness in cardiac surgery and to compare its effectiveness with propofol. The results that we obtained by recording BIS scores intra-operatively and by assessing patients postoperatively for awareness with recall using the Brice Questionnaire revealed that dexmedetomidine was as effective in reducing awareness and recall compared to propofol. Hence, from our study, we conclude that dexmedetomidine can also be used as an alternative sedative agent to prevent awareness and recall in cardiac surgery. Nonetheless, large prospective studies are needed to confirm these results.
